# The Doyen of Hospital Secretaries

**Published:** 1901-07-20

**Authors:** 


### The Hospital

A Journal of

The Medical Sciences and Hospital Administration.

Vol. XXX.—No. 773.] Edited by Sir Henry Burdett, K.C.B., and Solomon C. Smith, M.D., M.R.C.P. [July 20, 1901.

### The Doyen of Hospital Secretaries.

The recent controversy in connection with the National Hospital for the Paralysed and Epileptic has exhibited the consequences which result from an absence of continuity in administration by the governors of a hospital. It is the function and privilege of a managing board of independent governors, by the strict discharge of their duties, to support the responsible officials and shield them from unreasonable criticism. The abler the committee the stronger the official, because ability attracts ability and secures its application to the affairs of every properly conducted hospital. One of the saddest and least just consequences of what will probably be known in the history of our hospitals as the battle of Queen's Square, in the absence of a strong committee of governors who regularly attended its meetings, has been that the whole burden of defence has fallen practically upon the shoulders of the chief official. Anything more unreasonable or less desirable it is impossible to imagine. We hope that every hospital and kindred institution will take this lesson to heart, and that the governors of these charities will make it their business to ascertain definitely whether or not the affairs of each are managed by an active committee, and, in the latter alternative, to take immediate steps to strengthen the managerial body by the introduction of new blood in the person of energetic governors.

Mr. Burford Rawlings is universally recognised as the doyen of hospital secretaries. A man of intellectual vigour and considerable culture, he has for many years been one of the ablest writers on hospital subjects, and has rendered yeoman service on many occasions to the cause of the hospitals. Amongst those who have to do with the management of hospitals, especially in London, Mr. Rawlings has been rightly regarded as a man of sound judgment and wide knowledge, and his opinion has been sought on many occasions of difficulty to the advantage of those who have had the wisdom to seek his counsel. It is not too much to say that as a writer his work has been of a high order, and that much of it is likely to endure.

This is not the place to tell the origin of the National Hospital for the Paralysed. It is the place, however, to testify that for nearly thirty-five years Mr. Burford Rawlings has devoted the best of his energies and the whole of his time to the work of building up to its present standard this, the greatest of English hospitals for the paralysed. How absorbing and single-minded has been his interest in the work is proved by the knowledge that the emoluments which he has been paid are smaller than those received by gentlemen connected with other institutions where the work has not been nearly so onerous. Money has never been a consideration with Mr. Rawlings, who in the most self-denying way has put his heart into the work and has so succeeded in raising the average annual receipts from under £5,000 in 1865 to nearly £22,000 in 1897. In the former year the property in investments was under £13,000; to-day it is considerably over £200,000. The annual expenditure was about £1,700 when Mr. Burford Rawlings commenced his work; to day it is about £18,000 a year. The assured income of the hospital in 1866 was under £1,000; to-day it is upwards of £10,000. The total expenditure upon maintenance at the first date was under £7,000, and at the end of 1899 it had exceeded £295,000. The number of available beds was only 35 in 1866; they are now 200 in number. There is much more that we might say in connection with the actual results mainly due to the life-work of Mr. Burford Rawlings in regard to the National Hospital, as expressed in figures, but the figures we have given speak for themselves.

Mr. Burford Rawlings' work, thus briefly recorded, is most honourable to himself, and has been of inestimable advantage to a class of sufferers who were formerly much neglected to their injury. In the busy world of to-day brain and nerve affections are on the increase, and in no other city in the world is the strain which tends to produce those diseases greater than in London. For this reason all classes of the community, and especially the governors and patients of the National Hospital for the Paralysed, owe the deepest gratitude to Mr. Burford Rawlings, and we hope that, at any rate, his colleagues connected with the management of the Metropolitan hospitals who know his worth will organise a dinner at which expression may be given to the universal feeling of appreciation which his life-work has excited towards himself. No doubt Mr. Burford Rawlings like other men has made his mistakes, but whatever those mistakes may have been they are in fact relatively unimportant compared with the splendid results as we have briefly stated them by quoting facts which cannot be controverted. We hope that his experience and services may be secured for some large institution. Be this as it may we are confident that the governors of the National Hospital for the Paralysed will certainly grant a liberal pension to Mr. Burford Rawlings on his retirement.

